# Skewed X-inactivation is common in the general female population

**DOI:** 10.1038/s41431-018-0291-3

**Published:** 2018-12-14

**Authors:** Ekaterina Shvetsova, Alina Sofronova, Ramin Monajemi, Kristina Gagalova, Harmen H. M. Draisma, Stefan J. White, Gijs W. E. Santen, Susana M. Chuva de Sousa Lopes, Bastiaan T. Heijmans, Joyce van Meurs, Rick Jansen, Lude Franke, Szymon M. Kiełbasa, Johan T. den Dunnen, Peter A. C. ‘t Hoen, BIOS consortium, Bastiaan T Heijmans, Peter AC ’t Hoen, Joyce van Meurs, Dorret I Boomsma, René Pool, Jenny van Dongen, Jouke J Hottenga, Marleen MJ van Greevenbroek, Coen DA Stehouwer, Carla JH van der Kallen, Casper G Schalkwijk, Cisca Wijmenga, Sasha Zhernakova, Ettje F Tigchelaar, P Eline Slagboom, Marian Beekman, Joris Deelen, Diana van Heemst, Jan H Veldink, Leonard H van den Berg, Cornelia M van Duijn, Bert A Hofman, André G Uitterlinden, P Mila Jhamai, Michael Verbiest, H Eka D Suchiman, Marijn Verkerk, Ruud van der Breggen, Jeroen van Rooij, Nico Lakenberg, Hailiang Mei, Jan Bot, Dasha V Zhernakova, Peter van ’t Hof, Patrick Deelen, Irene Nooren, Matthijs Moed, Martijn Vermaat, René Luijk, Marc Jan Bonder, Maarten van Iterson, Freerk van Dijk, Michiel van Galen, Wibowo Arindrarto, Szymon M Kiełbasa, Morris A Swertz, Erik W van Zwet, Aaron Isaacs, Rick Jansen, Lude Franke, GoNL consortium, LC Francioli, A Menelaou, SL Pulit, F van Dijk, PF Palamara, CC Elbers, PB Neerincx, K Ye, V Guryev, WP Kloosterman, P Deelen, A Abdellaoui, EM van Leeuwen, M van Oven, M Vermaat, M Li, JF Laros, LC Karssen, A Kanterakis, N Amin, JJ Hottenga, EW Lameijer, M Kattenberg, M Dijkstra, H Byelas, J van Setten, BD van Schaik, J Bot, IJ Nijman, I Renkens, T Marschall, A Schönhuth, JY Hehir-Kwa, RE Handsaker, P Polak, M Sohail, D Vuzman, F Hormozdiari, D van Enckevort, H Mei, V Koval, MH Moed, KJ van der Velde, F Rivadeneira, K Estrada, C Medina-Gomez, A Isaacs, SA McCarroll, M Beekman, AJ de Craen, HE Suchiman, BA Hofman, B Oostra, AG Uitterlinden, G Willemsen, M Platteel, JH Veldink, LH van den Berg, SJ Pitts, S Potluri, P Sundar, DR Cox, SR Sunyaev, JT den Dunnen, M Stoneking, P de Knijff, M Kayser, Q Li, Y Li, Y Du, R Chen, H Cao, N Li, S Cao, J Wang, JA Bovenberg, I Pe’er, PE Slagboom, CM van Duijn, DI Boomsma, GJ van Ommen, PI de Bakker, MA Swertz, C Wijmenga

**Affiliations:** 1 Department of Human Genetics, Leiden University Medical Center, Leiden, The Netherlands; 2 Faculty of Bioengineering and Bioinformatics, Lomonosov Moscow State University, Moscow, Russian Federation; 3 Department of Biomedical Data Sciences, Leiden University Medical Center, Leiden, The Netherlands; 4 GenomeScan B.V. Leiden, Leiden, The Netherlands; 5 Department of Clinical Genetics, Leiden University Medical Center, Leiden, The Netherlands; 6 Department of Anatomy and Embryology, Leiden University Medical Center, Leiden, The Netherlands; 7 Department of Internal Medicine, ErasmusMC, Rotterdam, The Netherlands; 8 Department of Psychiatry, VU University Medical Center, Neuroscience Campus Amsterdam, Amsterdam, The Netherlands; 9 University of Groningen, University Medical Center Groningen, Department of Genetics, Groningen, The Netherlands; 10 Centre for Molecular and Biomolecular Informatics, Radboud Institute for Molecular Life Sciences, Radboud University Medical Center, Nijmegen, The Netherlands; 11 Department of Internal Medicine, Maastricht University Medical Center, Maastricht, The Netherlands; 12 Department of Gerontology and Geriatrics, Leiden University Medical Center, Leiden, The Netherlands; 13 Department of Neurology, Brain Center Rudolf Magnus, University Medical Center Utrecht, Utrecht, The Netherlands; 14 Genetic Epidemiology Unit, ErasmusMC, Rotterdam, The Netherlands; 15 Department of Epidemiology, ErasmusMC, Rotterdam, The Netherlands; 16 SURFsara, Amsterdam, The Netherlands; 17 Department of Medical Genetics, Center for Molecular Medicine, University Medical Center Utrecht, Utrecht, The Netherlands; 18 Department of Computer Science, Columbia University New York, New York, USA; 19 The Genome Institute, Washington University, St. Louis, MI USA; 20 Department of Forensic Molecular Biology, ErasmusMC, Rotterdam, The Netherlands; 21 Department of Evolutionary Genetics, Max Planck Institute for Evolutionary Anthropology, Leipzig, Germany; 22 Bioinformatics Laboratory, Department of Clinical Epidemiology, Biostatistics and Bioinformatics, Academic Medical Center Amsterdam, Amsterdam, The Netherlands; 23 Life Sciences Group, Centrum Wiskunde & Informatica, Amsterdam, The Netherlands; 24 Department of Human Genetics, Radboud University Medical Center Nijmegen, Nijmegen, The Netherlands; 25 Center for Neuroscience, Donders Institute for Brain, Cognition and Behaviour, Radboud University Nijmegen Medical Center, Nijmegen, The Netherlands; 26 Program in Medical and Population Genetics, Broad Institute of Harvard and MIT, Cambridge, MA USA; 27 Department of Genome Sciences, University of Washington, Seattle, WA USA; 28 Department of Clinical Genetics, ErasmusMC, Rotterdam, The Netherlands; 29 Rinat-Pfizer Inc., South San Francisco, CA USA; 30 Forensic Laboratory for DNA research, Leiden University Medical Center, Leiden, The Netherlands; 31 BGI-Shenzhen, Shenzhen, China; 32 BGI-Europe, Copenhagen, Denmark; 33 Legal Pathways Institute for Health and Bio Law, Aerdenhout, The Netherlands

**Keywords:** Population genetics, Genome informatics

## Abstract

X-inactivation is a well-established dosage compensation mechanism ensuring that X-chromosomal genes are expressed at comparable levels in males and females. Skewed X-inactivation is often explained by negative selection of one of the alleles. We demonstrate that imbalanced expression of the paternal and maternal X-chromosomes is common in the general population and that the random nature of the X-inactivation mechanism can be sufficient to explain the imbalance. To this end, we analyzed blood-derived RNA and whole-genome sequencing data from 79 female children and their parents from the Genome of the Netherlands project. We calculated the median ratio of the paternal over total counts at all X-chromosomal heterozygous single-nucleotide variants with coverage ≥10. We identified two individuals where the same X-chromosome was inactivated in all cells. Imbalanced expression of the two X-chromosomes (ratios ≤0.35 or ≥0.65) was observed in nearly 50% of the population. The empirically observed skewing is explained by a theoretical model where X-inactivation takes place in an embryonic stage in which eight cells give rise to the hematopoietic compartment. Genes escaping X-inactivation are expressed from both alleles and therefore demonstrate less skewing than inactivated genes. Using this characteristic, we identified three novel escapee genes (*SSR4*, *REPS2*, and *SEPT6*), but did not find support for many previously reported escapee genes in blood. Our collective data suggest that skewed X-inactivation is common in the general population. This may contribute to manifestation of symptoms in carriers of recessive X-linked disorders. We recommend that X-inactivation results should not be used lightly in the interpretation of X-linked variants.

## Introduction

X-chromosome inactivation is responsible for sex chromosome dosage compensation in females (XX), and ensures that X-chromosomal genes are not expressed at twice the levels of expression in males (XY) [1]. It occurs during early female embryonic development [2], but the exact timing in humans is still elusive. Once the choice for the inactivation of either the maternal or paternal X-chromosome is made, it is stably inherited to all daughter cells through mitosis. The choice of which of the two X-chromosomes is inactivated is random and does not depend on paternal or maternal origin. Therefore, females are mosaic and consist of a population of cells with preferential expression of either paternal or maternal X-chromosome. Not all females have equal proportions of cells with the paternal or maternal X-chromosome inactivated. This so-called skewed X-inactivation can be explained in different ways [3]. Firstly, skewing might be caused by selective pressure: a variant on one of the X-chromosomes is associated with lethality or limited survival and will undergo negative selection [4]. This explains, to a certain degree, symptoms in female carriers of variants associated with X-linked recessive diseases. For example, in the X-linked recessive disorder Duchenne muscular Dystrophy (DMD), a number of female cases with translocations that forced the inactivation of the normal *DMD* allele, were already reported in the 1980s [5–8]. Secondly, the cause of skewing may be purely stochastic in nature: just by chance more cells inactivate the paternal or maternal X-chromosome [9]. Given that X-inactivation is occurring in an embryonic stage where there are limited number of cells giving rise to the different germ layers, this may lead to skewing in the compartment arising from these limited sets of precursor cells.

X-chromosome inactivation is the example of an extraordinary epigenetic silencing mechanism spreading across the entire human ~160 Mbp chromosome. The inactive allele of the X-chromosome is heavily methylated, enriched for inactive histone modifications, and depleted for active ones [10]. X-inactivation requires a *cis*-acting master locus referred to as the X-inactivation center. This center is located in the long arm of X-chromosome in humans. It is known that expression of long non-protein coding RNA gene *XIST* within this center is essential for silencing [11, 12]. This gene is expressed only from the “inactive” X-chromosome and *XIST* RNA coats the inactive X allele [13].

The inactive X-chromosome is not entirely silent. In humans, nearly 15% of X-linked genes are thought to escape inactivation and are expressed from both active and inactive X-chromosomes [14]. The majority of these genes are located on the short arm of X-chromosome and form clusters [15]. The degree of “escape” from inactivation is variable between genes, tissues, time in development, and individuals. Thereby, X-linked genes could be classified as inactivated (that are silenced in all females), escape (escape inactivation in all females), and heterogeneous (escape X-inactivation in some females; also referred to as variable escapees) [14, 16]. Determining which genes escape X-inactivation has important clinical implications, as they may explain the inheritance pattern and/or penetrance of disease.

In this study, we investigated X-inactivation in the blood of a population of healthy daughters from the Genome of the Netherlands (GoNL) project [17] of which large-scale RNA-sequencing (RNA-seq) were generated. We took advantage of the availability of full genome sequences of the parents to unequivocally assign reads covering heterozygous single-nucleotide variants (SNVs) to the maternal and paternal alleles, and assessed the degree of skewing in the population and the genes that consistently escape X-inactivation in the population. Finally, we discuss the implications for the clinical diagnostic practice.

## Methods

### General

Figure S1 represents the schematic overview of the main set of procedures. Scripts and example data files are available at https://github.com/eshvetsova/X_inactivation_scripts

### Parent-of-origin assignment and allele-specific expression calling

Sample preparation and blood RNA-seq data processing have been described previously [17, 18] and in the Supplementary Methods. For the analysis of skewing patterns, we limited the analysis to the regions outside the pseudoautosomal regions (non-PAR regions) of the X-chromosome, to avoid mapping artifacts and influence of crossing-over events with the paternal Y-chromosome. We determined the parent of origin of all alleles heterozygous in the offspring by comparing offspring’s genotype information of each heterozygous loci with the corresponding genotype information of the parents. As males have only one variant of each SNV on X-chromosome, we assigned the allele equal to the one present in the father to be paternal and the remaining one to be maternal.

We extracted reads mapped to the SNV positions, separately for each individual. The reads were grouped by the presence of the reference or the alternative allele at the SNV position. Independently, the reads were grouped by their parent-of-origin allele based on the parental genotypes. Based on counts within these groups at each SNV position of an individual, we calculated the allelic ratio (${\mathrm{allelic}}\,{\mathrm{ratio}} = {\textstyle{{{\mathrm{alternative}}\,{\mathrm{count}}} \over {{\mathrm{alternative}}\, {\mathrm{count}} + {\mathrm{reference}}\,{\mathrm{count}}}}}$) and the paternal ratio (${\mathrm{paternal}}\,{\mathrm{ratio}} = {\textstyle{{{\mathrm{paternal}}\,{\mathrm{count}}} \over {{\mathrm{paternal}}\,{\mathrm{count}} + {\mathrm{maternal}}\,{\mathrm{count}}}}}$). We kept only SNV positions with coverage of at least 10 reads and only SNV positions overlapping exons of annotated genes. From the allelic and paternal ratios of these remaining SNVs, we calculated the mean and median paternal and allelic ratios for each individual.

### Analysis of individual genes

To determine whether a gene escapes X-inactivation, we selected skewed individuals with a median paternal ratio across the entire X-chromosome (see previous paragraph) of ≤0.35 or ≥0.65. We used the following procedure for the analysis of skewing status of individual genes:

Let *Mx* = median (paternal ratio[*i*,*k*]), where *i* = 1, …, *m*, with *m* being the number of SNVs (covered by ≥10 reads) on the X-chromosome in a sample and *Mg* = median (paternal ratio[*j*,*k*]), where *j* = 1, …, *n*, with *n* being the number of SNVs in a sample that are mapped to the gene *g* and where *k* = 1, …, *p*, with *p* being the number of samples with SNVs in the gene *g*. Note that *m* and *n* differ per sample and that we analyze only the genes with *p* ≥ 5. Further, let *Sx* = |*Mx* − 0.5| and *Sg* = |*Mg* − 0.5| be the skew factors (the distance from 0.5, where 0.5 reflects balanced expression of the paternal and maternal alleles), then for each gene we have two possible situations:

1. X-chromosome and gene *g* agree distance direction(*Mg* > 0.5 and *Mx* > 0.5)|(*Mg* < 0.5 and *Mx* < 0.5) => We perform the paired *t* test: *t*.test(*Sx*[*k*], *Sg*[*k*], alternative = “less”).
2. X-chromosome and gene *g* disagree on distance direction (*Mg* < 0.5 and *Mx* > 0.5)|(*Mg* > 0.5 and *Mx*  < 0.5) => We perform the paired *t* test: *t*.test(*Sx*[k], −*Sg*[*k*], alternative =“less”).

The null hypothesis in the test is that the median paternal ratio of the gene is not different from the overall median paternal ratio for that individual, consistent with absence of escapee behavior. The alternative hypothesis is that the median paternal ratio of the gene is closer to 0.5 than the overall median paternal ratio for that individual, consistent with escapee behavior.

### Analysis of mothers

RNA-seq data and DNA genotype for mothers in GoNL project were analyzed with the same quality controls and filters as applied to their offspring. Because of lack of information about parent of origin of alleles for mothers, we computed the measure of balance for each mother and offspring, as ${\mathrm{measure}}\,{\mathrm{of}}\,{\mathrm{balance}} = \frac{{\mathrm{min}}({\mathrm{alternative}}\,{\mathrm{count}},{\mathrm{reference}}\,{\mathrm{count}})}{{\mathrm{alternative}}\,{\mathrm{count}} + {\mathrm{reference}}\,{\mathrm{count}}}$. We calculated the median measure of balance for each individual as the median of measure of balance for all heterozygous SNVs with at least ten reads in the corresponding individual. Correlation between median measure of balance for mothers and their daughters was computed as Pearson's correlation coefficient.

### Simulation of skewing in the population after random X-inactivation

We ran simulations to demonstrate how random X-inactivation in 4, 8, 16, or 32 precursor cells would translate into different skewing patterns in the population. To this end, we used the rbinom function where *n* represents the number of cells, the number of trials equals 1, and the probability of the X-inactivation of the maternal X-chromosome is 0.5. We then calculated the average of maternal inactivation events across cells (equivalent to the paternal ratio) for each individual and this 10,000 times to arrive at a population distribution. We compared the theoretical distributions with the empirically observed distribution and used the Kolmogorov–Smirnov test to evaluate which theoretical distribution of paternal ratios was closest (highest *p* value in the test) to the empirical distribution.

### Ethics approval, consent, and data availability

The ethical approval for this study lies with the individual participating cohorts (CODAM, LL, LLS, and RS) and institutional review boards. A broad consent for participation in research, including research on genotypes, was obtained from all participants. Given the privacy-sensitive nature of the DNA and RNA data, the data have been deposited at the European Genome-Phenome Archive (EGA) under the accession number EGAS00001001077 and is under controlled access. Requests for the data can be filed in the EGA system and will be handled by the BIOS data access committee. The committee will provide access to researchers for studies with a solid scientific background.

Information on the variants that were used to call the novel escapee genes was submitted to LOVD and are publicly available at https://databases.lovd.nl/shared/individuals/00173710 until https://databases.lovd.nl/shared/individuals/00173724

## Results

### Overall characterization of the input data and methods

We have characterized the X-inactivation patterns in the blood of healthy individuals using RNA-seq data derived from 79 adult daughters from trios of the GoNL whole-genome sequencing project. The availability of parental DNA sequences makes it possible to accurately distinguish the maternal and the paternal chromosome. After filtering for non-exonic single-nucleotide variants (SNVs), SNVs with low coverage, and SNVs in pseudoautosomal regions, we obtained 30–150 informative, heterozygous SNVs per individual (Figure S2). We determined the parent of origin for each allele and calculated the allelic and paternal ratios. We define the allelic ratio as the ratio between the counts for the alternative allele and the total allele counts at that position, and the paternal ratio as the ratio between the counts for the paternal allele and the total allele counts at that position. We subsequently calculated the mean and median paternal ratio across the X-chromosome in each individual, as a measure for the degree of skewing of X-inactivation.

### Distribution of skewing in the population: examples of skewed and non-skewed individuals

Skewing in X-inactivation means preferential expression of the paternal (median paternal ratio more than 0.5) or maternal (median paternal ratio <0.5) chromosome. Non-skewed individuals have paternal ratios close to 0.5. Skewing in X-inactivation should not have a consistent effect on the allelic ratio, and the mean allelic ratios are expected to be close to 0.5 in each individual.

**Fig. 1:**
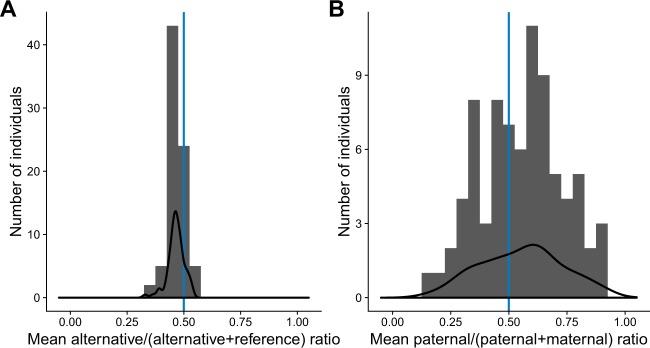
Distribution of mean allelic (**a**) and paternal (**b**) ratios for each individual. Black lines are the smoothed density curves corresponding with the obtained distributions

The distributions of the mean allelic and paternal ratios for the 79 daughters are shown in Fig. 1a, b, respectively. As expected, the mean allelic ratio has a narrow peak slightly shifted to the left relative to 0.5, which is likely attributed to reference bias [19]. The distribution of the paternal ratios is much wider. We identified 14 (=17.7%) individuals with preferential expression of maternal X-chromosome (median paternal ratio ≤0.35) and 25 (=31.6%) individuals with preferential expression of the paternal X-chromosome (median paternal ratio ≥0.65). These thresholds for skewed X-inactivation were defined as such, because there were no individuals with median allelic ratios beyond these threshold values (Fig. 1a). At the extreme end, we identified seven individuals with pronounced skewing towards the paternal or maternal chromosome in the blood (median paternal ratio ≥0.85 or ≤0.15) and two individuals with a median ratio of 1, effectively coming down to the inactivation of the same X-chromosome in all blood cells.

Examples of the distributions of the allelic and paternal ratios across all SNVs with sufficient coverage in an individual are presented in Figure S3. The degree of skewing did not depend on the age of the individual (Figure S4).

### Correlation between skewing in mothers and daughters

**Fig. 2:**
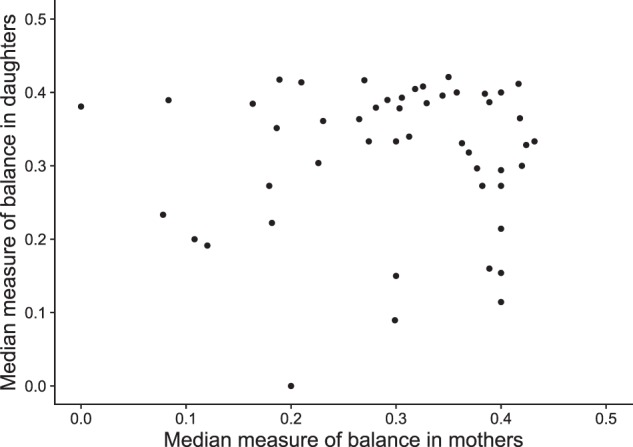
Lack of association between X-inactivation status in mothers and daughters. Scatter plot of median measure of balance for mothers (*x*-axis) and daughters (*y*-axis) at least ten reads coverage on heterozygous SNVs. There is no significant correlation (Pearson’s *ρ* = 0.038, *p* value = 0.7934)

If X-inactivation is a random process, we should not observe correlation in skewing between mothers and daughters. We checked this, but, as we do not know the parental origin of the alleles in the mothers, we calculated an alternative measure of balance, the median balance ratio (defined as the lowest of ${\mathrm{measure}}\,{\mathrm{of}}\,{\mathrm{balance}} = \frac{{{\mathrm{min(alternative}}\,{\mathrm{count}},{\mathrm{reference}}\,{\mathrm{count)}}}}{{{\mathrm{alternative}}\,{\mathrm{count}} + {\mathrm{reference}}\,{\mathrm{count}}}}$ across all X-chromosomal SNVs in an individual). RNA-seq data and DNA genotypes for 141 mothers passed quality control. We observed similar skewing distributions in the population of mothers as we found for the populations of daughters, and thus confirmed the presence of individuals with extremely skewed X-inactivation in the normal population (Figure S5). For the 49 complete mother:daughter pairs, we did not observe significant correlation between the skewing in mothers and daughters (Pearson's correlation coefficient 0.038, Fig. 2). These results imply that the imprinting status of a mother does not affect the inactivation status of her daughter, as expected from the random nature of the postconceptional X-inactivation process.

### Simulations of random X-inactivation

**Fig. 3:**
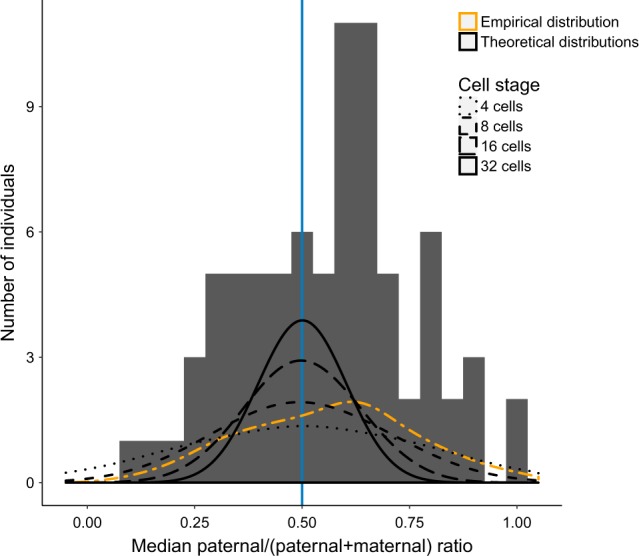
Theoretical assessment of cell numbers at the time of X-inactivation. Comparison of the empirical median paternal ratio distribution for heterozygous SNVs with more than ten reads per individual (orange line) with theoretical distributions under the hypothesis that X-inactivation takes place at the 4 (dotted black line), 8 (dashed black line), 16 (long dashed black line), and 32 (solid black line) precursor stage. Theoretical distribution at eight initial lineage-restricted precursor cells is most comparable with empirical distribution (highest *p* value = 0.011, two-sample Kolmogorov–Smirnov test)

Although the X-inactivation process is random, this does not imply that the expression of maternal or paternal chromosomes is equal in each individual. X-inactivation is an event in early embryonic development at a stage where there is only a limited number of precursor cells for the hematopoietic lineage present. To test how many blood (hematopoietic) precursor cells would be present at the time of X-inactivation to explain the degree of skewing observed in the general female population, we performed simulations. In case X-inactivation happens when there are four precursor cells present, it is quite likely that all of them, just by chance, inactivate the same (paternal or maternal) chromosome. One can see that 1 out of 16 individuals would express only the paternal X-chromosome and 1 out of 16 individuals would express only the maternal X-chromosome, that is, one out of eight individuals show complete skewing of X-chromosomal expression. When the initial pool consists of 32 cells, this chance is only approximately 5 × 10^−10^. We observed that the distribution of paternal ratios in the population, in a scenario where X-inactivation in the embryonic stage where eight cells give rise to the hematopoietic compartment, was most similar to the observed distribution in the female population under study (Fig. 3).

### *XIST* is expressed from the inactive X-chromosome

*XIST* is a long noncoding RNA, responsible for the initiation of X-inactivation. *XIST* is transcribed from a single X-chromosome poised for inactivation. Concordantly, in those individuals who contain SNVs in *XIST*, we observe that *XIST* is expressed from one chromosome and other X-linked genes from the opposite one (Figure S6A). When comparing *XIST*’s median paternal ratio with overall median paternal ratio for skewed individuals, we see that the distance of these ratios to 0.5 are opposite to the general pattern in almost all skewed individuals (Figure S6B). These observations confirm the expression of *XIST* from the inactive X-chromosome.

### Not all known escapee genes escape X-inactivation in blood

The presence of individuals with extreme skewing patterns allows us to analyze possible escape from X-inactivation for individual genes, as the escapee genes should demonstrate more balanced expression from the paternal and maternal chromosomes than the X-inactivated genes. For this analysis, we included individuals with either paternal or maternal skewing (defined as paternal ratio ≤0.35 or ≥0.65; 39 samples), and compared the median paternal ratio for that gene to the median paternal ratio of all heterozygous loci in the X-chromosome for each individual and evaluated whether that gene consistently deviated from the median ratio across all the individuals with sufficient coverage, using a one-sided *t* test.

**Fig. 4:**
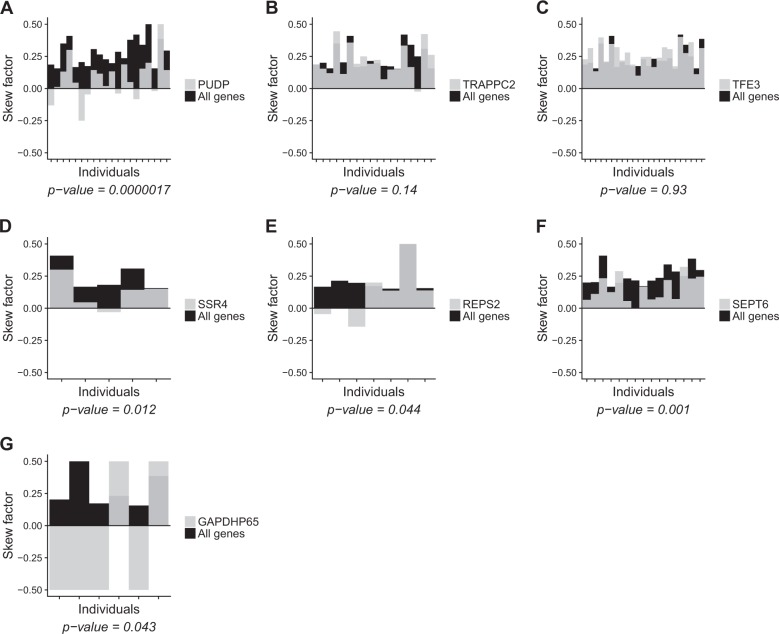
Assessment of escape from X-inactivation. Histogram of the skew factor for the entire X-chromosome (black bars) and for specific example genes (gray bars) in all skewed individuals (one bar for each individual) with coverage ≥10 on heterozygous SNVs in those genes. A one-sided test was used to test whether the ratios for a given gene were significantly different from the median ratio for the entire X-chromosome. In **a**, **b**, two “known” escapee genes: [14, 20–23] *PUDP* (ENSG00000130021) appears to escape X-inactivation, whereas *TRAPPC2* (ENSG00000196459) does not. In **c**–**g**, several genes not known to escape X-inactivation: **c** *TFE3* (ENSG00000068323) does not escape X-inactivation (in line with the literature), whereas *SSR4* (ENSG00000180879), *REPS2* (ENSG00000169891), and *SEPT6* (ENSG00000125354) were identified to escape X-inactivation for the first time in our study. **g** *GAPDH65* (ENSG00000235587) was found to be significant, but is a pseudogene and a likely false-positive gene due to inaccurate read mapping

**Fig. 5:**
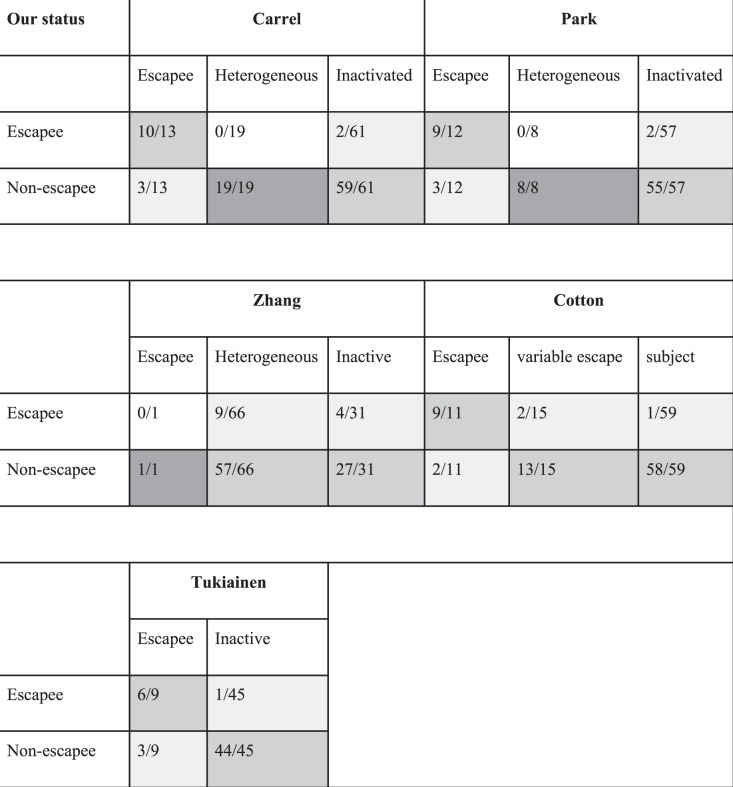
Overview of X-chromosomal genes that do (significant, *p* < 0.05) or do not (non-significant, *p* ≥ 0.05) escape X-inactivation in blood in our study in comparison to previous studies. Note: The first number in each cell corresponds to the number of escapee (significant, p < 0.05) or non-escapee (non-significant, p > =0.05) genes in our study, status of which matches the corresponding status in the literature [14,20–23]. The second number in each cell shows how many overlapping genes between our study and each of the referenced studies have the corresponding status according to the literature. Shading of the cells reflects degree of overlap (white: 0%, light grey: 1-50 %, grey: 51-99%, dark grey: 100%) Tissues analyzed: Carrel - primary human fibroblast cell lines, rodent/human somatic cell hybrids [14] Park - primary human fibroblast cell lines, rodent/human somatic cell hybrids [14] Zhang - immortalized human B-cells [22] Cotton - human fibroblast cell lines [20] Tukiainen - diverse human tissues [23]

Of 271 X-linked genes present in our data, 113 had SNVs with sufficient coverage in at least five individuals (informative genes). As expected, for most of the analyzed genes we do not see evidence for consistent escapee behavior, like for *TFE3* (Fig. 4d). We compared the results of our escapee behavior analysis with previous studies (Fig. 5). For the majority of “known” escapee genes, like *PUDP* (Fig. 4a), we obtained significant evidence for escape from X-inactivation in blood, but others, like *TRAPPC2* (Fig. 4b), do not show such evidence. Collectively, 21 of the informative genes were previously reported to escape X-inactivation in at least one study [14, 20–23], but only 11 of them escape X-inactivation in blood, according to our data. On the contrary, we found three genes that escape X-inactivation according to our data, *SSR4*, *REPS2*, and *SEPT6* (Fig. 4d–f), but have not been described as an escapee (*SSR4* and *SEPT6*) or have been reported to escape X-inactivation in a subgroup of individuals (*REPS2*) [20]. Another gene that appeared significant in our study is *GAPDHP65* (Fig. 4g), but this (pseudo)gene demonstrated a clear reference bias (for SNVs in this gene, only reference allele is expressed), likely due to mapping of reads derived from homologous genes, and should therefore not be regarded as a new escapee gene. Results for all 113 informative genes are presented in Supplementary Table 1. Collectively, we have identified several novel variable escapee genes and our data reveal that many “known” escapee genes are most probably variable escapees.

## Discussion

We report that skewed X-inactivation is common in the general female population. The degree of skewed X-inactivation reported earlier varies considerably [23, 24]. This is partly due to the differences in the assays used to assess the X-inactivation status. The most commonly used assay examines the DNA methylation status of the polymorphic *AR* locus (cf. HUMARA assay). Another group of assays analyzes allele-specific RNA expression at distinct heterozygous loci by quantitative reverse transcription-polymerase chain reaction (RT-PCR). The latter assays provide a more direct output measurement of X-inactivation. A direct comparison between allele-specific expression and HUMARA assay [25] demonstrated a number of inconsistencies and suggested that methylation status does not always reflect expression and that the HUMARA assay may be influenced by preferential amplification of *AR* alleles with shorter repeats. Moreover, expression of a single X-linked locus may not reflect the expression status of the entire X-chromosome as there are genes with variable levels of escape from X-inactivation in the healthy population. The combination of genome and RNA-seq-based analysis presented here can be regarded as an aggregate of all allele-specific expression measurements over the entire X-chromosome, and a robust and direct way of assessing X-inactivation status and skewing per individual. This makes it a useful clinical diagnostic tool for assessing X-inactivation status. In case the cost of RNA-seq are prohibitive, allele-specific quantitative RT-PCR assays could serve as an alternative, in particular when heterozygous loci have been identified by Sanger sequencing, gene panel, whole-exome sequencing, or whole-genome sequencing. However, based on the presented results, we strongly recommend not relying on single SNVs for the assessment of the X-inactivation status, but to at least include SNVs in several different genes.

Our current RNA-seq-based results stand out from previous papers, as we have observations along the entire X-chromosome and can uniquely assign each of these observations to the maternal or paternal chromosome, given the availability of the full parental haplotypes. Nevertheless, most of our results are consistent with earlier reports. In the largest study so far, Amos-Landgraf et al. [24] determined the distribution of X-inactivation patterns in blood samples from 1005 phenotypically unaffected newborn infants and adult women, using the *AR* methylation assay. In the resulting data set, 25% of the individuals demonstrated skewing ratios >0.7 or <0.3, and 8% of the individuals demonstrated ratios >0.8 or <0.2. X-inactivation ratio is normally distributed without mean shift. We observed very similar percentages (27 and 10%, respectively). In an earlier report by Sharp et al. [26], higher percentages were reported, possibly because of technical issues. Percentages were notably higher in elderly individuals. We have not observed a similar increase in skewing with age as reported [24, 26–30]. This may be partly attributable to differences in the age distribution studied, the assay used, the loci studied, or the tissue analyzed. It may also be that the relationship between methylation (used in these studies to assess skewing) and expression is gradually loosening with age. The observation that skewing does not increase with age in our population (age range 20–64 years) (Figure S4) argues against clonal expansion of hematopoietic cells as an explanation for the observed skewing pattern.

In a recently published RNA-seq-based paper from the GTEx consortium assessing X-inactivation in the general population across tissues, only 1 out of 449 individuals demonstrated extreme skewing (>95% across 16 tissues) [23], where we find already 2 in our population of 79 individuals. This may be partly explained by the fact that we are able to provide an accurate assignment of each allele to the paternal or maternal X, where parental genotypes are not available in the GTEx cohort. In the GTEx paper, it is nicely demonstrated that, despite variable escapee behavior across tissues, the X-inactivation patterns are usually consistent across tissues. Together with results from other studies [26, 31], this suggests that X-inactivation status in the blood is at least partly predictive for X-inactivation status in other tissues.

Assessment of the X-inactivation status has important implications for clinical diagnostics. Monoallelic or preferential expression of one of the alleles (skewing) is often seen as an indication of the presence of a nonsense mutation that induces nonsense-mediated decay. However, monoallelic expression needs to be seen in the context of the inactivation status of the entire X-chromosome. We show here that the mere fact that expression of only one allele is observed provides insufficient proof for its pathogenicity. This is further corroborated by the lack of correlation between the X-inactivation status of mothers and daughters, in line with the stochastic nature of the embryonic X-inactivation process. Proof for pathogenicity is only obtained when other (non-escapee) genes demonstrate biallelic expression. If this is not the case, the individual may just be a case of extreme skewing of X-chromosomal expression, which is also observed in the normal population. Knowledge of the X-inactivation status is also important for the classification of the increasing number of variants of unknown significance (VUS) identified by genome-wide sequencing technologies. Often, inheritance helps to classify VUS, but X-linked segregation patterns may be clouded by skewed X-inactivation. Skewing of X-inactivation may also explain the phenomenon of symptomatic female carriers of X-linked recessive disorders and differences in penetrance of dominant disorders. There have been a number of conflicting reports on the association of the X-inactivation status with clinical symptoms in these disorders [9, 32–36]. The assessment of X-inactivation status may explain why these relationships are difficult to consolidate: the frequently used *AR* methylation status may not be entirely predictive for the inactivation status of the disease locus. Moreover, the sole assessment of the *AR* methylation status does not tell whether the disease or the normal allele is preferentially inactivated in a given individual.

The dynamics of X-inactivation in humans are still largely unknown, but they are well studied in mice. Initiation of random X-inactivation starts in the inner cell mass mouse female blastocyst embryos at embryonic day (E)4, whereas imprinted X-inactivation occurs at day E2 and remains in the trophoblast cells [37, 38]. There are important differences between the mechanisms of X-inactivation in humans and mice. In humans, random X-inactivation has not been observed in the inner cell mass at least until day E7 and imprinted X-inactivation may not occur in the trophoblast cells [39–41]. Interestingly, *XIST* and another long noncoding RNA *XACT* are expressed from both X-chromosomes in blastocysts [41]. In our study, we simulated random X-inactivation to calculate the number of initial lineage-restricted blood precursor cells and demonstrate that the observed skewing patterns in the blood of the healthy female population are most consistent with X-inactivation in an embryonic stage where there are eight cells present that give rise to the hematopoietic compartment.

Previous studies [14, 20–23] reported numerous genes to escape from X-inactivation. However, results were not entirely consistent between studies. This can be attributed to differences in technical and statistical procedures, and differences in the tissues analyzed and the transcripts expressed from the genes in those tissues. Moreover, it appears that that there is heterogeneity regarding escapee genes between individuals, tissues, and time of development; those are the so-called variable escapees [16]. Figure S6B contains an illustration of this heterogeneous behavior: the paternal ratio for the *TRAPPC2* gene is close to 0.5 in one of the individuals with skewed X-inactivation, but very close to the median paternal ratio for all genes in most other individuals with similar degree of skewing, suggesting that it does not escape X-inactivation in the majority of individuals. We report here a small number of genes (14 in total), for which we established consistent escapee behavior in blood across the population.

In conclusion, we provide a robust and comprehensive view on the X-inactivation patterns observed in the general population and provide arguments for the need of careful assessment and interpretation of skewed X-inactivation in the clinical diagnostic practice.

## Electronic supplementary material

Supplemental Methods

Supplemental Figure and Table Legends

Supplemental Figure S1

Supplemental Figure S2

Supplemental Figure S3

Supplemental Figure S4

Supplemental Figure S5

Supplemental Figure S6

Supplemental Table S1
