# Supplementary material for: Skewed X-inactivation is common in the general female population
Source: Eur J Hum Genet. 2018 Dec 14;27(3):455–65. doi: 10.1038/s41431-018-0291-3 (PMC6460563; doi:10.1038/s41431-018-0291-3)
Supplement: Supplementary file 1 — Supplemental Methods [file 41431_2018_291_MOESM1_ESM.pdf]

Supplementary Methods

Study subjects

For the whole blood transcriptome analysis, we took individuals who were subjected to whole genome sequencing as part of the Genome-of-the-Netherlands (GoNL) project[1] and who were subjected to whole blood transcriptome analysis as part of the BBMRI-NL Biobank-based Integrative Omics Study (BIOS)[2,3]. The GoNL project sequenced 250 trios. From those, we included 79 female children (age distribution: 20 - 64, mean age = 36.2, median age = 33.3) with whole blood RNA-seq data passing all quality criteria and checked for genotype concordance with the whole genome sequencing data. The individuals were participants from one of four biobanks: LifeLines-DEEP (LLD), Leiden Longevity Study (LLS), Netherlands Twin Registry (NTR), Rotterdam Study (RS).

Sample preparation

Details are described in Zhernakova et al., 2017[3]. Total RNA from whole blood was deprived of globin using Ambion's GLOBIN clear kit and subsequently processed for sequencing using Illumina's Truseq version 2 library preparation kit. Paired-end sequencing of 2x50bp was performed using Illumina's Hiseq2000, pooling 10 samples per lane. Read sets per sample were generated using CASAVA, retaining only reads passing Illumina's Chastity Filter for further processing. Data was generated by the Human Genotyping facility (HugeF) of ErasmusMC (The Netherlands, see URLs).

RNA Seq data processing

Details are described in Zhernakova et al., 2017[3]. Initial QC was performed using FastQC (v0.10.1), removal of adaptors was performed using cutadapt (v1.1), and Sickle (v1.2) was used to trim low quality ends of the reads (minimum length 25, minimum quality 20). The sequencing reads were mapped to the masked human genome (HG19, all single nucleotide variants with allele frequency >0.01 in GoNL samples replaced by "N") using STAR (v2.3.125). To further reduce the influence of reference bias, WASP (version x)[4] was applied, using the genotypes in the vcf files from the matched genome sequences as an input, to remove reads that aligned to different positions after variant substitution. The gene definitions were based on Ensembl version 71.

Supplementary References

1. Genome of the Netherlands Consortium. Whole-genome sequence variation, population structure and demographic history of the Dutch population. *Nat Genet* 2014;46:818–825

2. Bonder, M.J., Luijk, R., Zhernakova, D.V *et al*. Disease variants alter transcription factor levels and methylation of their binding sites. *Nat Genet* 2017;49:131–138.

3. Zhernakova DV, Deelen P, Vermaat M *et al*. Identification of context-dependent expression quantitative trait loci in whole blood. *Nat Genet* 2017;49:139–145.

4. van de Geijn, B., McVicker, G., Gilad, Y., Pritchard, J.K. WASP: allele-specific software for robust molecular quantitative trait locus discovery. *Nat Methods* 2015;12:1061–1063.
