# Supplementary material for: Skewed X-inactivation is common in the general female population
Source: Eur J Hum Genet. 2018 Dec 14;27(3):455–65. doi: 10.1038/s41431-018-0291-3 (PMC6460563; doi:10.1038/s41431-018-0291-3)
Supplement: Supplementary file 2 — Supplemental Figure and Table Legends [file 41431_2018_291_MOESM2_ESM.docx]

# **Supplemental Data**

Supplementary Table S1: Escapee status of 113 informative genes and comparison with previous studies. Ensembl Gene IDs are from Ensembl release 71 and positions on the X-chromosome refer to the GRCh37 (hg19) genome build.

Supplementary Figure S1: Workflows used in the different analyses.

Supplementary Figure S2: Number of informative SNVs. Distribution of the number of heterozygous SNVs covered with at least ten reads per individual.

Supplementary Figure S3: Comparison of non-skewed and skewed individuals. Examples of allelic (left) and paternal (right) ratios for heterozygous SNVs with more than ten reads for the individual in an individual with no skewed X-inactivation (A) and with skewed X-inactivation (B). The histograms in panel A is symmetric relative to 0.5 and has no extreme values close to 0 or 1. The allelic ratios in panel B range from 0 to 1 and are bimodally distributed, but all paternal ratios are close to 1, indicating the only the paternal X-chromosome is expressed.

Supplementary Figure S4: Lack of association of skewing status with age of the individual. Scatter plot of the absolute median paternal ratio (x-axis) and age of individuals (y-axis) for all 446 heterozygous SNVs with enough coverage. There is no significant correlation (Pearson’s ρ*=*0.022, p-value = 0.8573).

Supplementary Figure S5: Observed skewing in mothers and daughters. Distributions of median measure of balance for mothers (A) and for daughters (B).

Supplementary Figure S6: *XIST* is expressed from the inactive X-chromosome. (A) Example of a scatter plot of the paternal (x-axis) and maternal (y-axis) counts for each heterozygous SNV with more than ten reads of the *XIST* gene (ENSG00000229807, blue triangles) and other genes (black dots) in a skewed individual. (B) Histogram of the skew factor for the entire chromosome (black bars) and for *XIST* gene (blue bars) in skewed individuals (each individual in a different bar).
