# Supplementary figures and images for: Skewed X-inactivation is common in the general female population
Source: Eur J Hum Genet. 2018 Dec 14;27(3):455–65. doi: 10.1038/s41431-018-0291-3 (PMC6460563; doi:10.1038/s41431-018-0291-3)

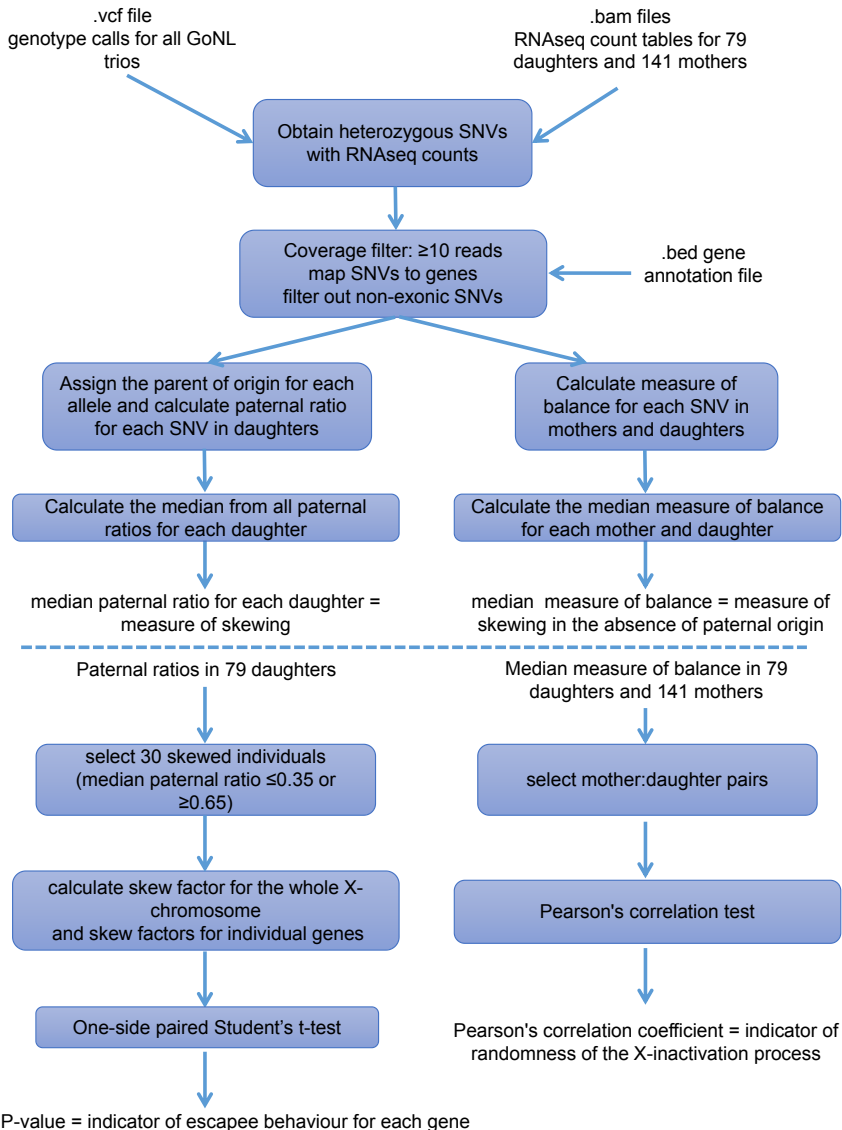

Supplement: Supplementary file 3 — Supplemental Figure S1 [file 41431_2018_291_MOESM3_ESM.pdf]

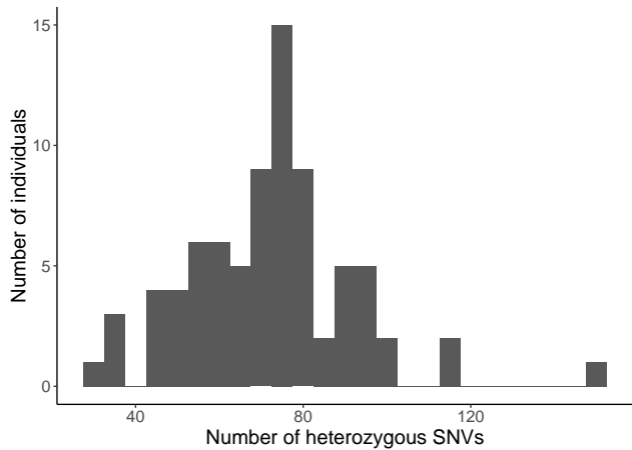

Supplement: Supplementary file 4 — Supplemental Figure S2 [file 41431_2018_291_MOESM4_ESM.pdf]

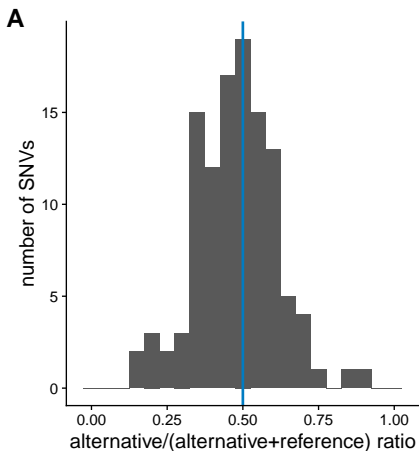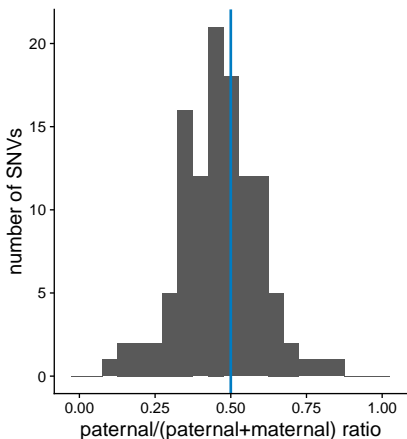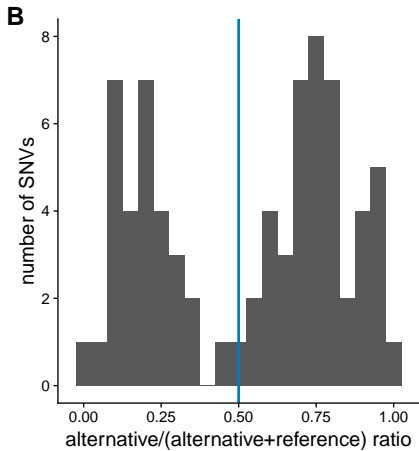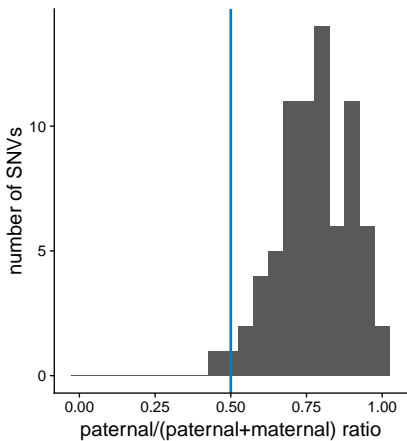

Supplement: Supplementary file 5 — Supplemental Figure S3 [file 41431_2018_291_MOESM5_ESM.pdf]

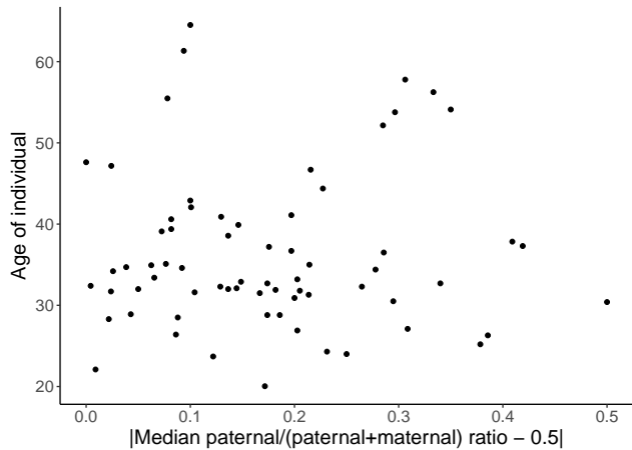

Supplement: Supplementary file 6 — Supplemental Figure S4 [file 41431_2018_291_MOESM6_ESM.pdf]

**A**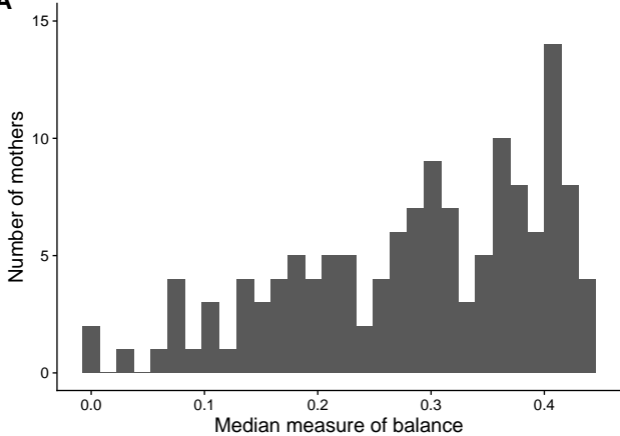**B**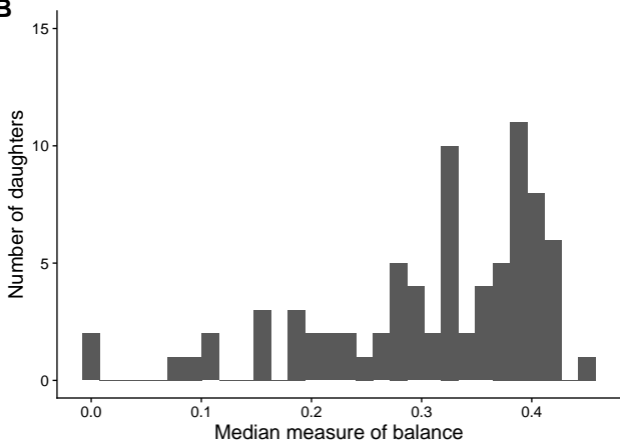

Supplement: Supplementary file 7 — Supplemental Figure S5 [file 41431_2018_291_MOESM7_ESM.pdf]

**A**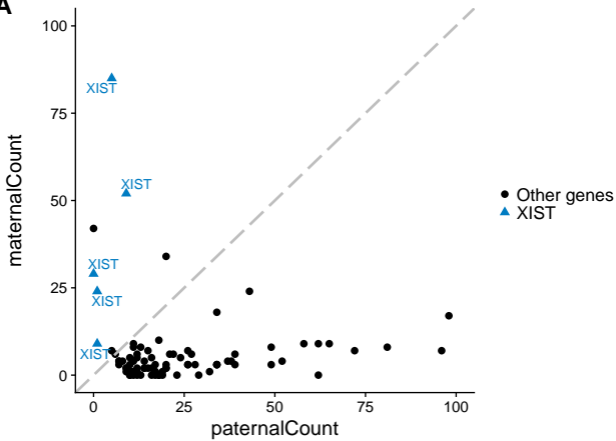**B**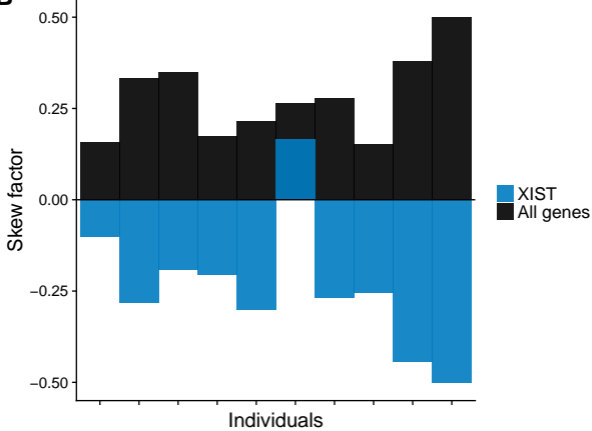

Supplement: Supplementary file 8 — Supplemental Figure S6 [file 41431_2018_291_MOESM8_ESM.pdf]
